# A Machine Learning Application Based in Random Forest for Integrating Mass Spectrometry-Based Metabolomic Data: A Simple Screening Method for Patients With Zika Virus

**DOI:** 10.3389/fbioe.2018.00031

**Published:** 2018-04-11

**Authors:** Carlos Fernando Odir Rodrigues Melo, Luiz Claudio Navarro, Diogo Noin de Oliveira, Tatiane Melina Guerreiro, Estela de Oliveira Lima, Jeany Delafiori, Mohamed Ziad Dabaja, Marta da Silva Ribeiro, Maico de Menezes, Rafael Gustavo Martins Rodrigues, Karen Noda Morishita, Cibele Zanardi Esteves, Aline Lopes Lucas de Amorim, Caroline Tiemi Aoyagui, Pierina Lorencini Parise, Guilherme Paier Milanez, Gabriela Mansano do Nascimento, André Ricardo Ribas Freitas, Rodrigo Angerami, Fábio Trindade Maranhão Costa, Clarice Weis Arns, Mariangela Ribeiro Resende, Eliana Amaral, Renato Passini Junior, Carolina C. Ribeiro-do-Valle, Helaine Milanez, Maria Luiza Moretti, Jose Luiz Proenca-Modena, Sandra Avila, Anderson Rocha, Rodrigo Ramos Catharino

**Affiliations:** ^1^Innovare Biomarkers Laboratory, School of Pharmaceutical Sciences (FCF), University of Campinas, Campinas, Brazil; ^2^RECOD Laboratory, Institute of Computing (IC), University of Campinas, Campinas, Brazil; ^3^Department of Genetics, Evolution, Microbiology and Immunology, Biology Institute, University of Campinas, Campinas, Brazil; ^4^Campinas Department of Public Health Surveillance, Campinas, Brazil; ^5^São Leopoldo Mandic Institute and Research Center, Campinas, Brazil; ^6^Clinical Pathology Department, School of Medical Sciences, University of Campinas, Campinas, Brazil; ^7^Obstetrics and Gynecology Department, School of Medical Sciences, University of Campinas, Campinas, Brazil

**Keywords:** Zika virus, Zika diagnosis, diseases diagnosis, high resolution mass spectrometry, machine learning, random forest, feature importance, diagnosis classifier

## Abstract

Recent Zika outbreaks in South America, accompanied by unexpectedly severe clinical complications have brought much interest in fast and reliable screening methods for ZIKV (Zika virus) identification. Reverse-transcriptase polymerase chain reaction (RT-PCR) is currently the method of choice to detect ZIKV in biological samples. This approach, nonetheless, demands a considerable amount of time and resources such as kits and reagents that, in endemic areas, may result in a substantial financial burden over affected individuals and health services veering away from RT-PCR analysis. This study presents a powerful combination of high-resolution mass spectrometry and a machine-learning prediction model for data analysis to assess the existence of ZIKV infection across a series of patients that bear similar symptomatic conditions, but not necessarily are infected with the disease. By using mass spectrometric data that are inputted with the developed decision-making algorithm, we were able to provide a set of features that work as a “fingerprint” for this specific pathophysiological condition, even after the acute phase of infection. Since both mass spectrometry and machine learning approaches are well-established and have largely utilized tools within their respective fields, this combination of methods emerges as a distinct alternative for clinical applications, providing a diagnostic screening—faster and more accurate—with improved cost-effectiveness when compared to existing technologies.

## Introduction

Zika virus (ZIKV) is an emerging pathogen that belongs to the Flaviviridae family and, as with other members, ZIKV is classified as an arthropod-borne RNA virus (arbovirus). The association between ZIKV and microcephaly in newborns from the recent outbreak of this viral infection in South America has raised much concern in the medical community, especially for the significant amount of cases of microcephaly in potentially endemic areas (Enfissi et al., 2016; Schuler-Faccini et al., 2016), as well as for the demonstrated tropism of ZIKV for neural cells medical community, especially for the significant amount of cases of microcephaly in potentially endemic areas (Attar, 2016; Cao-Lormeau et al., 2016; Enfissi et al., 2016). Furthermore, increasing evidence on the potential of ZIKV transmissions through contaminated blood products for transfusion shines an entirely different light over infection routes, broadening transmission sources beyond the mosquito bite (Musso et al., 2014; Motta et al., 2016).

These cases reinforce the importance of accurate ZIKV identification in a broad scope, ranging from newborn screening to the control of hemoderivatives. Additionally, since ZIKV can easily be clinically mistaken by other infections of similar symptomatic profile (To et al., 2015), bioanalytical approaches that accurately differentiate these conditions are vitally important.

Current laboratory diagnostic tests are still limited in accuracy, either because of cross-reactivity, as in the case of serological tests (Morizono and Chen, 2014; Fauci and Morens, 2016; Steinhagen et al., 2016), or because of the current lack of standardization/validation and sensitivity/specificity data, as is the case of reverse-transcriptase polymerase chain reaction (RT-PCR) (Eltzov et al., 2010). Furthermore, current analysis techniques demand a substantial amount of time to produce results (Pardee et al., 2016), and costs associated with kits, reagents, and specialized personnel per sample run are considerably high (Rouet et al., 2005), especially considering that endemic areas are located in regions of low-income and/or poor healthcare support (Fauci and Morens, 2016). Thus, there is great interest in providing an expeditious approach that can produce accurate results in a timely fashion and with a cost-effective workflow.

Mass spectrometry-based metabolomics has been widely utilized as a relevant alternative for diagnostic purposes in biological samples (Kind et al., 2016; Takayama et al., 2016; Deng et al., 2017), and data processing tools and spectral databases are key players in the success of these approaches (Gromski et al., 2015; Vinaixa et al., 2016), since the mass spectra of a given set of complex matrices reveals a multitude of chemical entities/molecules. This richness of information is the starting point for many comparative studies, for example, in the analysis of biological samples from individuals with a pathophysiological condition versus a control group with healthy individuals (Melo et al., 2017). By using data processing tools to drive this comparison, it is possible to establish which is the specific spectral signature for that particular condition based on their intrinsic differences, even if very subtle (Eiras et al., 2014). Such differences allow us to infer that spectral data of that particular sample group will behave, therefore, as a “fingerprint,” where feature by feature will compose a unique model of pattern recognition (Lima Ede et al., 2015).

Given the large amount of spectral data generated, and the requirement of always providing a comparison to obtain spectral signatures of the condition under study, bioinformatics approaches have been built to solve these problems, so that the classification/taxonomy of sample groups may be achieved (Johnson et al., 2015). In turn, machine learning (ML) approaches have allowed the comparison between spectral data of a large number of samples and sample groups (*N*), as opposed to a limited amount of data as in the case of multivariate data analysis (Zheng et al., 2014). Since ML models can be continuously fed with more information, it allows the user to focus only on the chemical species that provide actual discrimination between samples/sample groups (Smith et al., 2014; Acharjee et al., 2016).

The main objective of using ML in the method presented in this paper is to generate a classifier based on mass spectral input data from blood serum to predict, with high accuracy and precision, whether a patient is positive or negative for a disease, in this case, for the ZIKV infection. The mass spectral data of each sample (*m/z* value × intensity) is used as the input for all analyses and predictions performed herein.

For this purpose, we selected the Random Forest supervised ML algorithm (Breiman, 2001), which is nd used in many applications, e.g., image analysis (Shotton et al., 2013), cancer diagnosis (Suna et al., 2017), and genetic assignment (Sylvester et al., 2017). Random Forest is based on decision trees (Caruana and Niculescu-Mizil, 2006; Criminisi et al., 2012) and a probabilistic interpretation of its principles can be found in Murphy (2012). This ML algorithm has the following advantages when processing the data we have at hand:

- *High-classification performance*: Random Forest is one of the best classifiers for different problems (Fernández-Delgado et al., 2014).
- *No need of kernel and complex parametrization adjustments*: Random Forest is known as a non-parametrized method, which means it does not require a complex search of parameters, kernel transformation, neither is it sensitive to normalization of input data. Only two parameters are subject to adjust for performance tuning: number of feature randomly selected in each tree building cycle, which is commonly set to the root square of the number of input variables, and the number of trees in the forest, which is usually subject to simple grid search approach.
- *Execution performance*: A trained Random Forest classifier is a set of binary trees, which can be seen as a sequence of “if then else” statements being extremely fast at prediction time.
- *Feature importance*: Decision tree classifiers provide information about the relevance of each feature in the decision trees by evaluating how a change or omission of one feature impacts classification results. This is referred to as Out-of-Bag (OOB) evaluation concept used as a performance measurement in Breiman (1996) and further applied to Random Forest feature importance determination by the mean decrease of accuracy of OOB samples with features randomly permuted (Breiman, 2001; Altmann et al., 2010; Louppe et al., 2013). Importance assessment is a key property of the classification algorithm to provide explainability and accountability of results achieved by the classifier.

In this work, we rely upon feature importance analysis to rank and to isolate the most discriminant features generating a high-performance classifier, which identifies the presence (or not) of signatures of ZIKV in the patient’s mass spectral sample. Those ranked most discriminant features can also be used to single out some physical molecules, which are part of the signature and can be found with high presence in the serum positive patient’s blood in contrast with negative ones. This fact corroborates with physical evidence, the power of the method, which is in line with a new frontier in ML techniques called accountable or interpretable ML (Diakopoulos et al., 2017).

In summary, we propose an innovative methodology based on high-resolution mass spectrometry (HRMS), combined with the Random Forest algorithm (Breiman, 2001), to provide an accurate prediction model for discriminating serum samples of individuals with ZIKV. Since supervised methods, such as Random Forest induce classifiers (i.e., a set of features that provide a “fingerprint” for the viral infection), this model is intended to be employed as a fast and accurate test for ZIKV infection in healthcare institutions. With specificity and sensitivity over 95%, in addition to the relatively low cost per sample run, this novel platform shows potential for forming a large integrated database for further epidemiological studies in infections by ZIKV.

## Materials and Methods

### Ethics Statement

This study was conducted according to the principles expressed in the Declaration of Helsinki and was approved by the Research Ethics Committee of the University of Campinas, under the number 053407/2016. A written informed consent was obtained from all patients prior to enrollment. All samples were obtained from the Clinical Hospital of the University of Campinas.

### Research Participants and Specimen Collection

In total, 203 patients were included in this study, regardless of age and gender, in two main groups: ZIKV and control. Group division considered patients’ retrospective laboratory results, obtained after testing with RT-PCR (Table 1).

**Table 1:** Summary of the specimens included in the study regarding demographic information, clinical conditions, and results from reverse-transcriptase polymerase chain reaction (RT-PCR) performed during the high viremia period.

	Zika virus (ZIKV) symptomatic and current infected	ZIKV 1 month after infection	Symptomatic, but not ZIKV	Symptomatic dengue RT-PCR+	Healthy, asymptomatic more than 30 days
RT-PCR	+	+	−	−	−
Positive/Negative	Positive	Positive	Negative	Negative	Negative

Demographics					

Male	27	23	48	25	6
Female	16	16	16	21	5
Total of specimens	43	39	64	46	11
Mean age (median)	33.23 (33)	32.85 (32.2)	32.53 (31)	33.21 (33)	32.76 (30)

The *ZIKV group* consists of 82 patients split into: (a) 43 adults with acute ZIKV infection (i.e., within the high-viremia period) confirmed by positive RT-PCR test, in association with clinical presentation (symptoms) compatible with ZIKV infection (i.e., fever, joint pain, conjunctivitis, and rash); and (b) 39 patients after 30 days of confirmed ZIKV infection by positive RT-PCR test (i.e., after the acute phase).

The *control group* contains the remaining 121 patients in which (a) 64 presented the same clinical symptoms as described above for ZIKV infection, but with a negative result for real-time RT-PCR test for ZIKV, (b) 46 patients with dengue virus infection confirmed by positive immunosorbent (ELISA) test, and (c) 11 healthy adults, i.e., asymptomatic individuals who did not present any signs of infection within 30 days prior to sample collection which, therefore, also presented a negative result in RT-PCR for ZIKV.

All RT-PCR were performed using RNA extracted from the serum of the analyzed patients.

Serum of patients was obtained from 10 mL of peripheral blood collected in dry tube after peripheral venipuncture. All samples were transported on ice within less than 6 h to the Laboratory for Study of Emerging Viruses at the Biology Institute of the University of Campinas, where they were processed and tested for ZIKV on real-time RT-PCR. Aliquots of serum were kept at −80°C until HRMS analysis.

### ZIKV Detection by Real-Time RT-PCR

RNA samples were extracted from 140 µL of serum and urine using the QIAamp Viral RNA Mini Kit (Qiagen, Hilden, Germany) following manufacturer’s instructions. Samples were tested by One-step TaqMan real-time RT-PCR (Taqman RNA to-CT, Applied Biosystems) for the presence of ZIKV genomes.

Zika virus detection was performed with primers and probes adapted from the original described by Lanciotti et al. (2008) (*ZIKV-F: 5′-CCGCTGCCCAACACAAG-3′; ZIKV-R: 5′-CCACTAACGTTCTTTTGCAGACAT-3′; ZIKV-P: 5′-FAM—AGCCTACCTTGACAAGCAGTCAGACACTCAA—BHQ1-3′*). Briefly, all reactions were performed in a final volume of 12.5 µL with 50 ng of RNA, 10 mM forward and reverse primers, 5 mM probe, and 6.25 µL of TaqMan master mix (Applied Biosystems, Foster City, CA, USA), using the following cycling algorithm: 48°C for 30 min, 95°C for 10 min, followed by 45 cycles of 95°C for 15 s, and 60°C for 1 min. All real-time RT-PCR were performed in duplicate.

### HRMS Preparation and Analysis

10 µL of serum samples were diluted to a final volume of 1 mL in a methanol/water solution (1:1) (solution 1). After homogenization, the sample was further submitted to a second dilution of 10 µL into a 0.1% solution of formic acid in methanol/water (1:1), to a final volume of 1 mL. All samples from each research participant were prepared in duplicates.

Samples submitted to HRMS were directly infused into an ESI-LTQ-XL Orbitrap Discovery instrument (Thermo Scientific, Bremen, Germany). Metabolic fingerprint data were acquired using a sample flow of 10 µL/min, capillary temperature of 280°C, 5 kV of source voltage, and sheath gas at 10 arbitrary units. In addition to the biological duplicates, analytical triplicates were performed for each sample. The acquisition was performed in the mass-to-charge ratio (*m/z*) range of 700–1,700, in the positive ion mode.

### ML Method

The decision-making method we propose here for ZIKV detection has the following macro steps:

1. *Data preparation*: For our study herein, data samples of positive (with ZIKV) and negative’s patients (without ZIKV condition) are normalized and randomly divided into two main partitions (***P_train_*** = 80% and ***P_test_*** = 20% of the patients). The partition with 20% of the data (referred to as ***P_test_***) is left untouched for the final blind test to evaluate the designed diagnosis classifier. This is done to avoid any kind of overfitting to the available data. The remaining partition with 80% of the data (referred to as ***P_train_***) is then used for training and validation tests in the process of determining most discriminant features for ZIKV detection. For reference, during training, a classifier is induced while during validation its performance on the validation set is checked.
  This process is iterative as we shall detail next. We further divide ***P_train_*** into two subsets ***P_fit_***, with 80% of the data in ***P_train_*** and ***P_val_***, with the remaining data in ***P_train_***. ***P_fit_*** is then used in the induction of the classifier (learning stage) and ***P_val_*** in its evaluation. To account for possible variations in the splitting of fitting and validation sets, we repeat this process 10 times (here referred to as rounds) and report average performance numbers for the validation set with the corresponding SD. A small SD means there is no high variation across patients in the learning process of the algorithm.
2. *Most discriminant features identification and ranking*: Fitting and validation cycles of Random Forest classifier are iteratively executed, reducing the vector length representing each patient on each cycle by discarding the least significant ranked features. The feature importance measure is obtained in each cycle using the Out-of-Bag (OOB) calculation for the training samples over the trained trees. By sorting features in decreasing order of importance, we generate the feature ranking, which is updated on each step for the remaining ones after discarding part of the features located in ranking tail. The best performance achieved in this step determines the spectral signature features kept for further processing.
3. *Generate the diagnosis classifier*: Upon selecting the most discriminative features, we proceed to train the final classifier by using only such selected features. This allows us to now induce a simplified, yet powerful, classifier with only a subset of the original features (in our case a few dozen rather than thousands of initial features per patient). To train the final classifier, data from all patients in ***P_train_*** is considered. Finally, the resulting classifier is tested with the blind-test data ***P_test_*** and the final performance numbers are reported.
4. *Values distribution analysis of the spectral signature features*: Although the previous step resulted in a final classifier trained with the most important features to detect ZIKV, we take a step further to determine which of the selected best features have higher prevalence in the serum of positive patients. For that, we analyze the range of values of spectral signature features in positive and negative data samples. We refer to such features as ***marker*** (outstanding for the positive class) features. Probability distribution functions for the positive and negative values are compared using equality hypothesis test and higher values cumulative probability comparison.
5. *Marker features mapping into molecules*: The *m/z* values for the marker features are then mapped into physical molecules using the mass spectral techniques to corroborate evidences on the spectral mass signature used by the diagnosis classifier.

### Data Preparation

In the data preparation step, we normalize the input *m/z* × intensity vectors of the samples using the relative intensity of each vector (we divided all vector elements by their maximum value), as defined in the equations given below. The normalization is needed to work with a more well-defined range of values for the features and is standard procedure in ML.

$$F=\left[f_{\mathrm{i, j}}\right],\;f_{\mathrm{i, j}}=\frac{z_{\mathrm{i, j}}}{\max(z_{\mathrm{i, j}=1:k})}$$

$$L=\left[l_{i}\right],\;l_{i}=\{-1,+1\},\;(\mathrm{vector label})$$

$$M=[m_{j}],\;m_{j}=m/z\;\mathrm{value}\;(\mathrm{feature label})$$

where ***F*** comprises the measurements for all patients. Each row ***f*** ∈ ***F*** represents data measurements from one patient. As each patient has five different sets of measurements (replicates) to account for possible variations ***F*** has 1,015 feature vectors. Each feature vector of a patient, comprises some 10,000 *m/z* values, many of which are missing upon different measurements.

As previously mentioned, ***F*** is divided into ***P_train_*** and ***P_test_*** and this latter set is left untouched for the final test of the developed classifier. It is important to mention that all splitting procedures are done so that all replicates of a patient are put in the same partition—therefore, the splitting is always performed per patient and not per feature vector.

### Number of Trees Determination Using Grid Search Approach

For experiments described in this article, we used the default of square root of the total number of input features for the randomly selected features in each tree construction cycle, and the number of trees was defined by maximum between 40 and the square root of the total number of input variables. It is important to notice that during the reduction process, using this formula, the number of trees varies in each step according to the vector length.

**Figure 1:**
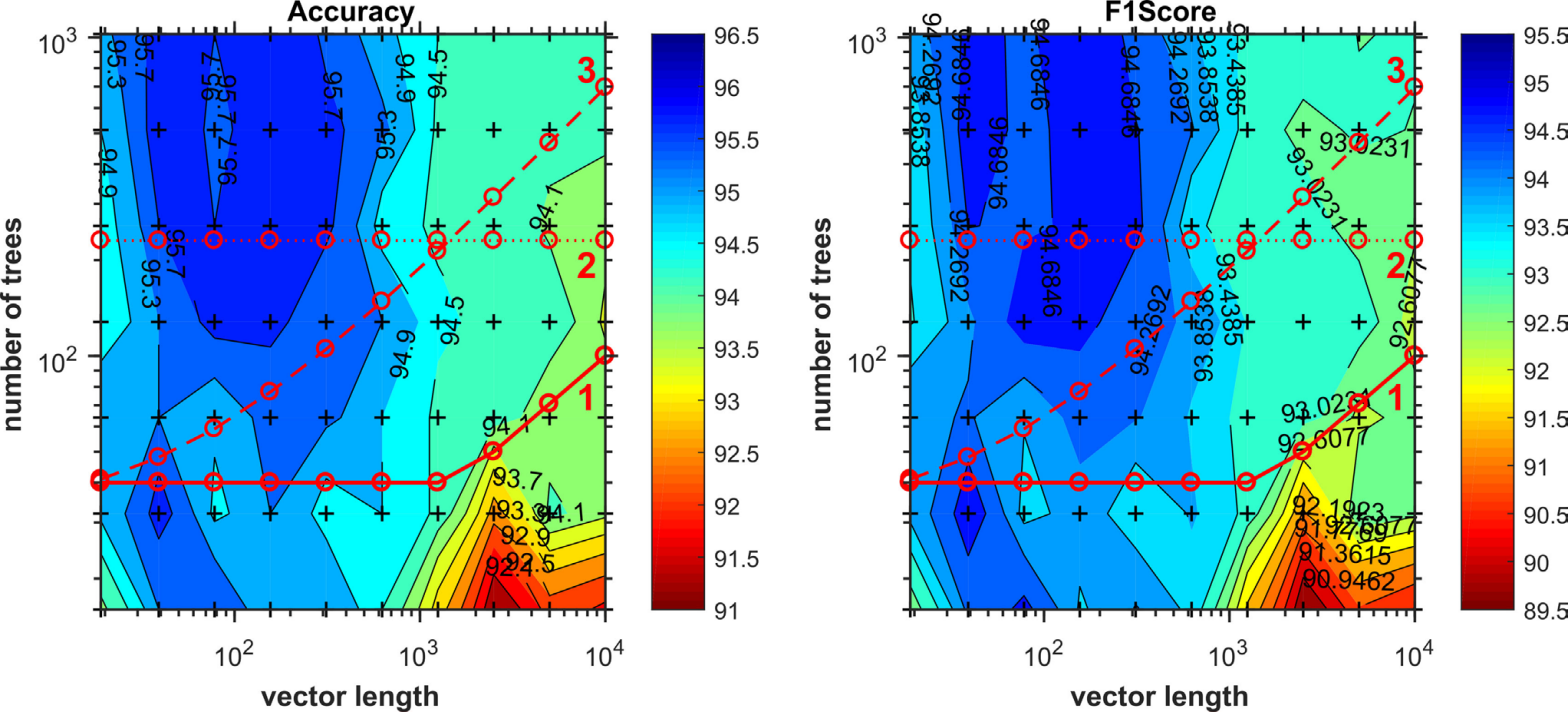
Number of trees given by grid search as function of vector length. Cross marks inside the chart denotes values evaluated during the grid search. Lines 1, 2, and 3 correspond to functions as expressed in Table 2 used to compute the number of trees on the evaluation of discriminant features reduction.

To select the number of trees used in the experiments, we performed a grid search varying the length of the ranked feature vectors, ranking them during the grid-search process, and the number of trees for each vector length from 1,024 to 16. By dividing two in each step, it generates a logarithmic grid, which could be plotted in the form of a contour surface, which colors regions delimited by isometric lines built from the grid *z*-axis values (we use accuracy and also f1score), generating the chart shown in Figure 1. By analyzing the regions of best achieved accuracy for validation, we selected three functions to determine the number of trees. The first was to use the initially determined default described above nt = max[40, sqrt(len)], second a constant value crossing the regions of good accuracy nt = 230, and the third one as function that crosses the chart diagonally.

Comparing the validation results of the most discriminant features process using each of the options above, as Table 2 shows, we opted to use the number of trees as nt = max[40, sqrt(len)], because all three final results are statically comparable, and although the Eq. 3 provides the smallest SD, the first choice runs much faster than the others, providing also the smallest number of signature features.

**Table 2:** Comparison of the most discriminant 10-round training and validation results using the three selected equations for the number of trees in each iteration as function of the ranked vector length.

Number of trees equation (ν = vector length)	Max[40,sqrt(ν)]		230		32 + [log2(ν)/2.sqrt(ν)]	
		
	Grid chart line 1		Grid chart line 2		Grid chart line 3	
		
	μ	σ	μ	σ	μ	σ
			
Best vector length	42		59		93	
Accuracy	96.54%	3.58%	96.03%	2.61%	96.12%	2.00%
Sensitivity	97.74%	3.66%	97.74%	3.66%	96.99%	3.71%
Specificity	95.34%	5.23%	94.31%	5.81%	95.26%	3.79%
Precision	93.99%	6.29%	92.82%	6.46%	93.66%	4.61%
NPV	98.46%	2.50%	98.55%	2.34%	98.02%	2.31%
F1Score	95.74%	4.23%	95.03%	3.17%	95.18%	2.42%
F1Neg	96.82%	3.38%	96.26%	2.78%	96.55%	1.78%

Green	Metric’s best value					
Rose	Metric’s worst value					

### Ranking Most Discriminant Features

The objective of this step is to discover which features carry most information for the separation of positive and control (negative) patients. This is carried out through a ranking approach in which less relevant features are eliminated iteratively. By repeating the fitting and validation process of the Random Forest with fewer features in each step, the rank for the top features is refined, and the impact on the overall classification metrics is measured in the validation set.

In each iteration, the rank of remaining ranked features is updated using the descending order of the mean of the 10 feature importance vectors stored in each training round. Only the portion of the rank corresponding to the ranked length processed in the iteration is updated, the tail remains with the upper discarded rank.

We evaluate the feature importance for each classifier through the out-of-bag calculation, which estimates the impact of a missing feature in the classification trees. To reduce the number of considered features in each step, we multiply the dimensionality (number of features) of feature vector ***f*** by a factor 0 < γ < 1, retaining only the ⌊|***f***’| × ***γ***⌋ most important features to the next step, where |⋅| measures the number of features in vector ***f***. This process is repeated until convergence—either by achieving a minimum set performance or when there is no feature to discard anymore. We determine the most discriminant features by the maximization of the classification performance metrics, e.g., using F1score as the measure to maximize, and we call them spectral signature features. We shall define such measures later in this paper.

### Generate Diagnosis Classifier

At this stage, we train the final diagnosis classifier using the most important features found in the previous step and all training data available in ***P_train_***. Afterward, we test the classifier using blind-test ***P_test_*** and report final results for ZIKV detection.

### Distribution Analysis to Find Marker Features

In addition to generating a ZIKV classifier—which can identify patients with the disease—we set forth the objective of determining which metabolites appear with higher intensity on the positive patients than in the control group.

By relying on the ranges of values of each selected feature using our Random Forest classifier, we can identify dependencies between features which results in a good separation for the two classes of interest. As we are looking for features with the highest values, we are interested only in the ones which can be analyzed in isolation without further dependencies on other features. For that, values distribution analysis is performed comparing the features probability distribution functions, seeking the ones with higher values in the positive samples than in the set of negative ones. We refer to such features as ***marker features*** for the disease, or simply marker features.

First, we apply an equality test to determine whether each feature has distinguishable distributions; if they are equal, we cannot test for the higher value condition. For this purpose, we use the two-sample Kolmogorov–Smirnov (KS) test (Massey, 1951; Miller, 1956) over the two discrete probability functions, $p(y),\;q(\bar{y})$, respectively, for ***y*** values of a feature in the spectral signature on positive patient’s samples and $\bar{y}\;$ values for same feature in the control group. After the equality hypothesis of KS test could not be confirmed, we apply the rule expressed in Equation Δ***_j_*** to identify marker features. It means that for a marker feature, the probability to find a value over the median of that feature in the set of positive patients is β higher than finding the same order of values in the set of negative patients. For instance, by setting β to 40% means that over the median of positive samples, we will find only 10% of negative samples

$$M_{j}∋M\;\mathrm{is a positive feature to disease},\;\mathrm{if}:$$

$$\Delta_{j}=\int_{\mathrm{median}(y_{j})}^{\max(F_{j})}q(\bar{y_{j}})-p(y_{j})>\beta$$

where *y_j_* is a *F_j_* value for a positive patient; $\bar{y_{j}}$ is a *F_j_* value for a negative patient; *p*(*y_j_*) is the probability distribution function of positive patients, and $q(\bar{y_{j}})$ the probability distribution function of negative patients; *P*(*y_j_*) is the cumulative distribution function (CDF) of *y* values, and $Q(\bar{y_{j}})$ is the CDF of $\bar{y_{j}}$; 0 < β < 0.5 CDF difference over median of the feature *j* for the positive patients (e.g., β = 40%).

## Results and Discussion

**Figure 2:**
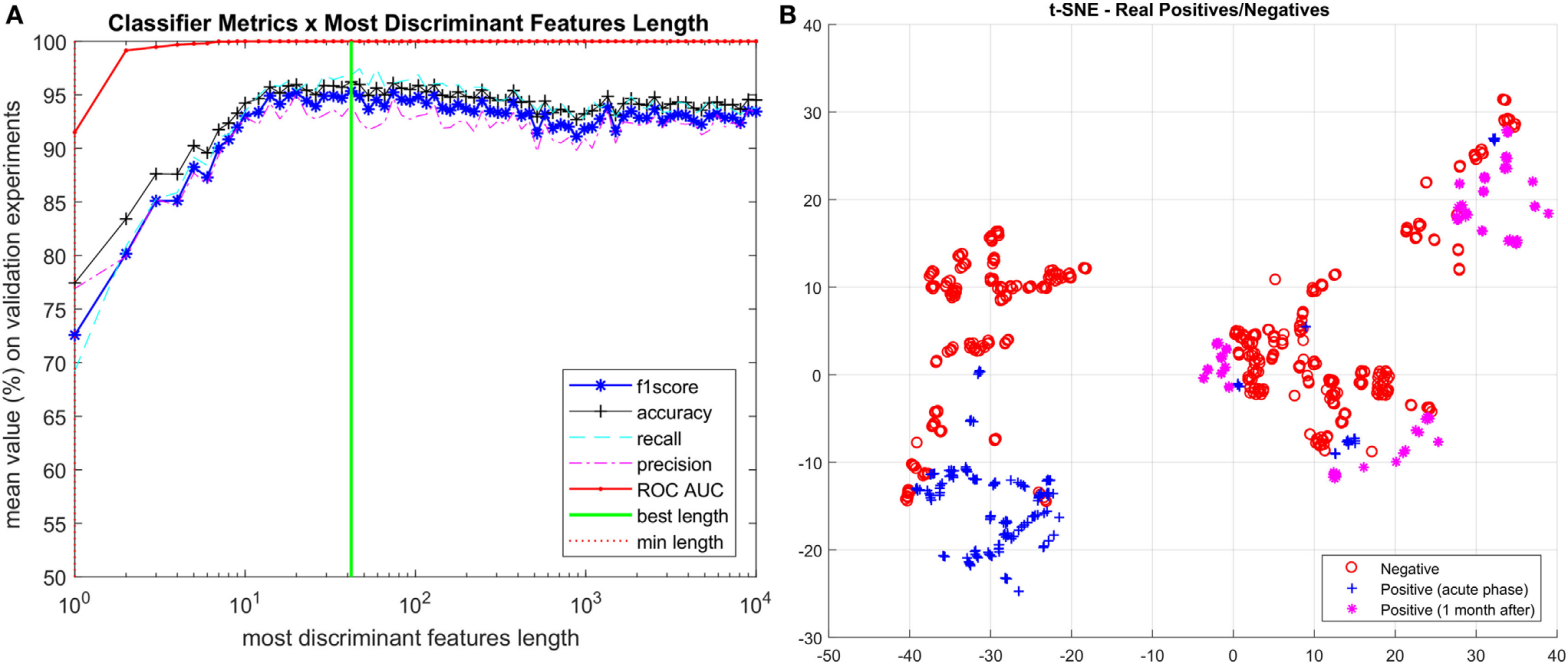
**(A)** Iterative process to determine the most discriminant ranked features. **(B)** Visualization of vectors with spectral signature features (length 42) using t-SNE technique. Vectors corresponding to positive Zika virus-infected patients are separated into two categories: acute phase and 1 month after infection.

**Figure 3:**
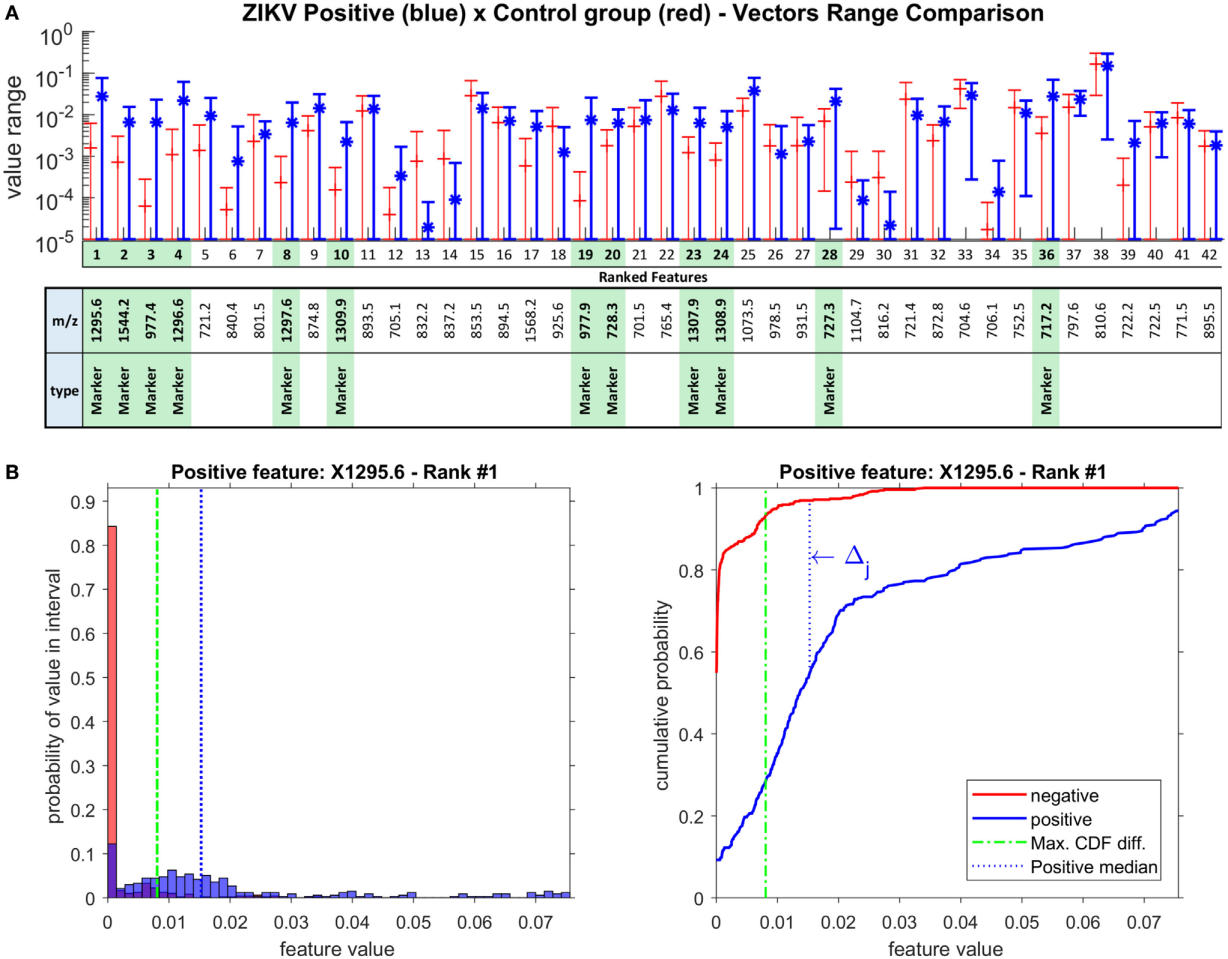
**(A)** Ranked features SD range in log scale for Zika virus (ZIKV) positive and control group (negative) vectors. The green highlight identifies the marker features for ZIKV, selected using the rationale of Δ_j_ > 40%. **(B)** Example of probability distribution and cumulative distribution charts for the main ranked feature for ZIKV, ion *m/z* 1,295.6 (Ganglioside); the rationale for Δ_j_ calculation is given on the right chart.

The iteration of reducing feature vector length and ranking most discriminant features is summarized in Figure 2A, starting with 10,000 features and shrinking by a factor of 0.9, we finally identified **42** features, listed in Figure 3A as the spectral features signature.

This is a remarkable result, as it allowed us not only to reduce the initial noisy 10,000 *m/z* measurements per patient to just 42 most discriminant for ZIKV virus, but also because it was the first time that the acute phase of ZIKV was accurately evaluated with patients 30 days after infection (i.e., non-acute phase). Thus, in order to visualize such features, we further projected them onto a 2D space through the *t*-distributed stochastic neighbor embedding (t-SNE) (Maaten and Hinton, 2008) visualization technique for high dimension data resulting in the chart shown on Figure 2B. Although just using 2 dimensions out of the 42 selected as important for classification, we can see a very good separation between ZIKV and control group samples. We also split the positive group into the two categories of ZIKV infected patients, the acute phase samples and the 1 month after infection samples. As we can see, most of the acute phase is grouped into a consistent cluster on the left side of the chart while the 1-month infected cases are spread into 3 other consistent clusters. The relation between the positive and negative samples in each region can also be addressed by the reduced vector analysis pointing out which sample belongs to each group and which ions they have in common. Ultimately, we envision the TSNE chart analysis being useful to identify which ions are present in each cluster giving a physical clue about what those clusters have in common further advancing the study toward more accountable models. This can be pursued in a future work.

Table 3 presents the average results for the validation set over the 10 rounds of training and validation along with the correspondent standard deviation, and the final numbers for the blind test. As expected, the blind test results are within the predicted range determined on the validation tests, and confirm the remarkable results achieved by the proposed technique.

**Table 3:** Zika virus (ZIKV) diagnosis classifier’s tests results.

Metric	Formula	10 rounds Validation tests		Blind final test
		Mean	σ	
Feature vector length		42		42
Real positives	*P* = *TP* + *FN*			15
Real negatives	*N* = *TN* + *FP*			24
Predicted positives	*TP* + *FP*			15
Predicted negatives	*TN* + *FN*			24
True negatives	*TN*			23
False positives	*FP*			1
False negatives	*FN*			1
True positives	*TP*			14
Accuracy	$\mathrm{ACC}=\frac{\left(\mathrm{SEN}+\mathrm{SPC}\right)}{2}$	96.54%	3.58%	94.49%
Sensitivity	$\mathrm{SEN}=\frac{\mathrm{TP}}{\mathrm{TP}+FN}$	97.74%	3.66%	93.33%
Specificity	$\mathrm{SPC}=\frac{TN}{TN+FP}$	95.34%	5.23%	95.65%
Precision	$\mathrm{PRC}=\frac{TP}{TP+FP}$	93.99%	6.29%	93.33%
Negative Predicted value	$\mathrm{NPV}=\frac{TN}{TN+FN}$	98.46%	2.50%	95.65%
F1Score	$F1S=2\cdot \frac{\mathrm{SEN}.\mathrm{PRC}}{\mathrm{SEN}+\mathrm{PRC}}$	95.74%	4.23%	93.33%

The chart on Figure 3A shows the logarithmic SD range for all 42 selected spectral signature features, identifying 12 markers for ZIKV, which are highlighted in green. The distribution analysis for the 42 spectral signature features was performed over all feature vectors as defined by Equation Δ***_j_***, using β = 40%. For illustration, Figure 3B brings the distribution histogram of the first ranked feature (*m/z* = 1,295.6) and the rationale of Δ_***j***_ calculation.

This group of 12 markers can be grouped by their *m/z* proximity, composing four groups of correlated cations: (1,295.6, 1,296.6, 1,297.6), (727.3, 728.3), (1,307.9, 1,308.9, 1,309.9), (977.4, 977.9), and two other individual cations: 1,544.2 and 717.2. This grouping occurs due to the chemical interpretation of the results; while these values are treated as independent variables among themselves, chemically, these features show an important correlation. For instance, in the group of values 1,295.6, 1,296.6, and 1,297.6, the biomarker is actually only 1,295.6, as the other two masses correspond to the natural isotopic distribution of carbon (i.e., ^13^C and ^14^C in the molecule). The same is true with all other groups, where the most relevant ion is that with the lowest nominal mass. It is noteworthy that this also occurs with divalent cations, as in the case of the group composed by 977.4 and 977.9, where the 1 Da difference is divided by 2 (*m/z*, where *z* = 2). This is an extremely important characteristic of mass spectra that provides even more reliability to the results, as this proves that the employed model effectively provided features/molecules that are discriminant of that particular group; since in the dataset these variables are completely independent, our results bring an outcome that is coherent chemically. Thus, the group of 12 marker features corresponds to 6 actual molecules, i.e., biomarker candidates.

After metabolomics database search, all six features were elucidated and identified as a pentapeptide (717.2) and a tetrapeptide (727.3, 728.3), a divalent (977.4, 977.9) and a monovalent ganglioside (1,295.6, 1,296.6, 1,297.6), a cardiolipin (1,307.9, 1,308.9, 1,309.9), and a bisphosphoglycerol (1,544.2500), which are the physical evidence of the positive ZIKV samples.

### Computing Performance Metrics

All experiments were performed using a Samsung 500R5H-XD3BR, Intel Core i7-5500CPU @ 2.40 GHz, 2 Cores, 4 logical processors, 8 GB of physical memory, 1 TB HD 5400RPM SATA-III 6GB/s. Programs were written in MATLAB script language and ran on MATLAB R2017a 64-bit version 9.2.0.538062. All ML algorithms and analyses in the end-to-end process from data preparation to distribution analysis take about 15 s per patient in the training (considering the five different measurements per patient). The time to analyze a feature vector of a patient at testing time is less than a second.

### Comparing Random Forest Classifier With Other Classifiers

Table 4 shows Random Forest compared with the well-known classification algorithm SVM using two different optimization algorithms: SMO (Sequential Minimal Optimization) and ISDA (Iterative Single Data Algorithm), and with a decision tree classifier, also with two different split criteria: GDI (Gini’s diversity index) and DEVIANCE (maximum deviance reduction, also known as cross entropy). The 10-round training and validation tests were executed over the 1,000 features full spectra vectors and also for the 42 signature features selected by the feature importance supervised reduction method. In short, RF performs best not only in the original complete feature space, but also on the selected best features thus justifying its use.

**Table 4:** Comparison of 10-round training and validation results between classifiers using same datasets for the full-length input vectors and for the signature features selected by the reduction method proposed in the article.

	SVM						Tree			
	Sequential minimal optimization		Iterative single data algorithm		Random forest		Gini’s diversity index		Deviance	
					
	μ	σ	μ	σ	μ	σ	μ	σ	μ	σ
**Vector length 10,000 (full spectra)**										

Accuracy	90.16%	5.96%	90.84%	6.28%	94.19%	3.59%	89.62%	5.60%	90.07%	5.91%
Sensitivity	87.88%	11.58%	89.25%	9.62%	94.06%	4.81%	87.41%	9.37%	88.83%	8.10%
Specificity	92.44%	7.58%	92.44%	7.58%	94.31%	5.25%	91.83%	5.47%	91.31%	4.23%
Precision	89.74%	10.05%	89.58%	10.34%	92.45%	6.29%	88.32%	7.41%	87.42%	6.22%
NPV	92.54%	6.78%	93.24%	6.05%	95.93%	3.13%	91.59%	5.89%	92.31%	5.42%
F1Score	88.08%	7.50%	89.00%	7.73%	93.11%	4.21%	87.54%	6.49%	88.08%	6.91%
F1Neg	92.17%	4.59%	92.63%	5.14%	95.03%	3.27%	91.59%	4.41%	91.79%	4.63%

**Vector length 42 (signature features)**										

Accuracy	93.13%	2.80%	93.42%	4.05%	96.54%	3.58%	91.22%	3.54%	91.24%	4.60%
Sensitivity	93.93%	5.10%	92.45%	5.20%	97.74%	3.66%	89.60%	3.62%	89.60%	6.28%
Specificity	92.34%	5.02%	94.39%	5.66%	95.34%	5.23%	92.84%	5.01%	92.89%	4.86%
Precision	89.91%	6.34%	92.39%	7.47%	93.99%	6.29%	89.87%	6.56%	89.81%	7.14%
NPV	95.89%	3.07%	94.93%	3.40%	98.46%	2.50%	92.83%	2.68%	92.92%	4.25%
F1Score	91.65%	3.26%	92.24%	4.84%	95.74%	4.23%	89.64%	4.31%	89.57%	5.60%
F1Neg	93.98%	2.62%	94.58%	3.66%	96.82%	3.38%	92.78%	3.32%	92.84%	3.74%
Green	Metric’s best value										
Rose	Metric’s worst value										

## Conclusion

The developed screening strategy using HRMS to assess ZIKV infection detects a set of 42 features, which are a spectral signature identified by a Random Forest classifier. 12 out of 42 features have high presence in the blood of patients due to ZIKV infection. This set of markers was validated using a powerful combination of statistical tools and are further supported by result comparison with those obtained with the current method for ZIKV diagnosis, RT-PCR. We hereby demonstrated that the combination between HRMS and the Random Forest algorithm is a robust platform that can be implemented in large-scale routine laboratories for rapid and straightforward detection of ZIKV, whether in patient screening or, as more recently recommended by the FDA, in donated blood and derivatives for transfusion. This approach is a work in progress, which will be the basis for the creation of a large database on molecules produced during ZIKV infection. This may lead to revealing new information on epidemiology, immunity, and pathogenesis of the ZIKV infection.

Due to the nature of the method and outstanding results achieved with ZIKV experiments, it is possible to envision that this method is a breakthrough technique in disease diagnosis tests.

Using our proposed platform, we envision that classifiers for many diseases can be developed. The only condition is that the serum of patients with the disease must contain information detected by the mass spectrometer; then, ML algorithms take care of extracting discriminative fingerprint for the condition of interest. Our aim is that, with one set of biofluids from any given patient with an unknown disease, we can submit such samples to multiple classifiers simultaneously, with a fast and reliable response to potential diagnostics.

## The Zika-Unicamp Network

**Albina Altemani**, Pathology Department, School of Medical Sciences, University of Campinas, Campinas, Brazil; **Ana Carolina Coan**, Neurology Department, School of Medical Sciences, University of Campinas, Campinas, Brazil; **Ana Caroline de Souza Barnabé**, Department of Genetics, Evolution and Bioagents, Biology Institute, University of Campinas, Campinas, Brazil; **Ana Lucia Rodrigues da Soledade**, Department of Genetics, Evolution and Bioagents, Biology Institute, University of Campinas, Campinas, Brazil; **Ana Paula de Moraes**, Department of Genetics, Evolution and Bioagents, Biology Institute, University of Campinas, Campinas, Brazil; **Andrea Paula Bruno von Zuben**, Campinas Department of Public Health Surveillance, Campinas, Brazil; **Carla Cristina Judice**, Department of Genetics, Evolution and Bioagents, Biology Institute, University of Campinas, Campinas, Brazil; **Daniel Augusto de Toledo Teixeira**, Department of Genetics, Evolution and Bioagents, Biology Institute, University of Campinas, Campinas, Brazil; **Évellyn Ribeiro de Morais**, Department of Genetics, Evolution and Bioagents, Biology Institute, University of Campinas, Campinas, Brazil; **Felipe Rebelo Santos**, Department of Genetics, Evolution and Bioagents, Biology Institute, University of Campinas, Campinas, Brazil; **Giuliane Jesus Lajos**, Obstetrics and Gynecology Department, School of Medical Sciences, University of Campinas, Campinas, Brazil; **Glaucia Maria Pastore**, Faculty of Food Engineering, University of Campinas, Campinas, Brazil; **João Renato Bennini Júnior**, Obstetrics and Gynecology Department, School of Medical Sciences, University of Campinas, Campinas, Brazil; **Juliana Almeida Leite**, Department of Genetics, Evolution and Bioagents, Biology Institute, University of Campinas, Campinas, Brazil; **Kleber Yotsumoto Fertrin**, Clinical Pathology Department, School of Medical Sciences, University of Campinas, Campinas, Brazil; **Leonardo Cardia Caserta**, Department of Genetics, Evolution and Bioagents, Biology Institute, University of Campinas, Campinas, Brazil; **Márcia Teixeira Garcia**, Clinical Pathology Department, School of Medical Sciences, University of Campinas, Campinas, Brazil; **Marco Aurélio Ramirez Vinolo**, Department of Genetics, Evolution and Bioagents, Biology Institute, University of Campinas, Campinas, Brazil; **Marcos Tadeu Nolasco da Silva**, Pediatric Immunology, Center for Investigation in Pediatrics, School of Medical Sciences, University of Campinas, Campinas, Brazil; **Maria Francisca Colella-Santos**, Department of Human Development and Rehabilitation, School of Medical Sciences, University of Campinas, Campinas, Brazil; **Maria Laura Costa**, Obstetrics and Gynecology Department, School of Medical Sciences, University of Campinas, Campinas, Brazil; **Roseli Calil**, Faculty of Food Engineering, University of Campinas, Campinas, Brazil; **Rosemeire Florênça de Oliveira de Paula**, Department of Genetics, Evolution and Bioagents, Biology Institute, University of Campinas, Campinas, Brazil.

## Ethics Statement

This study was conducted according to the principles expressed in the Declaration of Helsinki and was approved by the Research Ethics Committee of the University of Campinas, under the number 053407/2016. A written informed consent was obtained from all patients prior to enrollment. All samples were obtained from the Clinical Hospital of the University of Campinas.

## Author Contributions

CM, MD, JD, AA, and CA performed mass spectrometry experiments. CM, LN, SA, and AR conceived and executed the ML method. CM and LN wrote the manuscript. DO, TG, CE, EL, MM, MR, RR, and KM performed data analysis. PP, GM, GN, FC, CA, and JP-M processed serum samples and performed all molecular biology experiments. AF, RA, MR, EA, RJ, CR-V, HM, and MM performed patient recruitment, biofluids management, and clinical support. CM, LN, DO, SEFA, AR, and RC performed manuscript proofreading and prepared tables and figures. RC idealized all experiments and managed the research group. The Zika-Unicamp Network is mentioned as an initiative from the University of Campinas of mutual collaboration in the Brazilian Plan for Fighting Zika Virus.

## Conflict of Interest Statement

The authors declare that the research was conducted in the absence of any commercial or financial relationships that could be construed as a potential conflict of interest. The reviewer HW and handling Editor declared their shared affiliation.
